# Chronic Behavioral Manipulation via Orally Delivered Chemogenetic Actuator in Macaques

**DOI:** 10.1523/JNEUROSCI.1657-21.2021

**Published:** 2022-03-23

**Authors:** Kei Oyama, Yukiko Hori, Yuji Nagai, Naohisa Miyakawa, Koki Mimura, Toshiyuki Hirabayashi, Ken-ichi Inoue, Masahiko Takada, Makoto Higuchi, Takafumi Minamimoto

**Affiliations:** ^1^Department of Functional Brain Imaging, National Institutes for Quantum Science and Technology, Chiba 263-8555, Japan; ^2^Systems Neuroscience Section, Primate Research Institute, Kyoto University, Inuyama, Aichi 484-8506, Japan; ^3^Japan Science and Technology Agency, PRESTO, Kawaguchi, Saitama, 332-0012, Japan

**Keywords:** chemogenetics, DREADDs, monkey, nonhuman primates, PFC, working memory

## Abstract

The chemogenetic technology referred to as designer receptors exclusively activated by designer drugs (DREADDs) offers reversible means to control neuronal activity for investigating its functional correlation with behavioral action. Deschloroclozapine (DCZ), a recently developed highly potent and selective DREADD actuator, displays a capacity to expand the utility of DREADDs for chronic manipulation without side effects in nonhuman primates, which has not yet been validated. Here we investigated the pharmacokinetics and behavioral effects of orally administered DCZ in female and male macaque monkeys. Pharmacokinetic analysis and PET occupancy examination demonstrated that oral administration of DCZ yielded slower and prolonged kinetics, and that its bioavailability was 10%-20% of that in the case of systemic injection. Oral DCZ (300-1000 μg/kg) induced significant working memory impairments for at least 4 h in monkeys with hM4Di expressed in the dorsolateral prefrontal cortex (Brodmann's area 46). Repeated daily oral doses of DCZ consistently caused similar impairments over two weeks without discernible desensitization. Our results indicate that orally delivered DCZ affords a less invasive strategy for chronic but reversible chemogenetic manipulation of neuronal activity in nonhuman primates, and this has potential for clinical application.

**SIGNIFICANCE STATEMENT** The use of designer receptors exclusively activated by designer drugs (DREADDs) for chronic manipulation of neuronal activity for days to weeks may be feasible for investigating brain functions and behavior on a long time-scale, and thereby for developing therapeutics for brain disorders, such as epilepsy. Here we performed pharmacokinetics and *in vivo* occupancy study of orally administered deschloroclozapine to determine a dose range suitable for DREADDs studies. In monkeys expressing hM4Di in the prefrontal cortex, single and repeated daily doses significantly induced working-memory impairments for hours and over two weeks, respectively, without discernible desensitization. These results indicate that orally delivered deschloroclozapine produces long-term stable chemogenetic effects, and holds great promise for the translational use of DREADDs technology.

## Introduction

The chemogenetic tool referred to as DREADDs (designer receptors exclusively activated by designer drugs) has been widely used for controlling neuronal activity and animal behavior in many species, including rodents and nonhuman primates (Roth, 2016). By combining mutated muscarinic receptors (excitatory hM3Dq and inhibitory hM4Di) with specific actuators, such as clozapine-N-oxide (CNO), these chemogenetic techniques offer a means to remotely manipulate the activity of a specific neuronal population for minutes to hours (Urban and Roth, 2015). In addition to the acute control, repeated administration of the actuators enables chronic chemogenetic manipulation of neuronal activity for days to weeks (Grace et al., 2016; Nation et al., 2016; Wakaizumi et al., 2016; Andreoli et al., 2017; Yu and Münzberg, 2018; Desloovere et al., 2019; Paretkar and Dimitrov, 2019; Goossens et al., 2021), and thus has potential therapeutic applications. For example, chronic and reversible inhibition of neuronal activity can be an effective means for seizure control and epilepsy treatment (Lieb et al., 2019). For this purpose, oral delivery of agonists seems to be suitable, as it typically produces relatively long-lasting effects compared with systemic (i.e., intramuscular and intravenous) injections (Karbwang et al., 1997). In addition, oral administration is noninvasive and relieves the subject from stress during chronic treatment. It is also beneficial for neuroscience research aimed at long-term control of neuronal activity in freely moving animals without restraint or stress. However, recent studies have reported that CNO has low permeability into the brain, and that its metabolite, clozapine, activates not only DREADDs but also endogenous receptors and transporters, leading to potential off-target effects (Gomez et al., 2017; Tran et al., 2020). To circumvent this issue, alternative selective DREADDs agonists have been explored and validated (Bonaventura et al., 2019; Weston et al., 2019; Nagai et al., 2020).

Deschloroclozapine (DCZ) is one of the third-generation agonists for muscarinic DREADDs that represents high permeability into the brain and is highly potent and selective to DREADDs compared with prior agonists, such as CNO and C21 (Nagai et al., 2020). Importantly, low doses of DCZ exert chemogenetic effects with rapid onset that last several hours in both rodents and nonhuman primates (Nagai et al., 2020). This indicates that DCZ is a promising chemogenetic actuator that can be used for a wide variety of objectives (e.g., Hirabayashi et al., 2021; Hori et al., 2021; Oyama et al., 2021). In particular, its superior efficacy and brain permeability permit its use for oral administration. Indeed, it has been demonstrated that orally delivered DCZ effectively induces behavioral changes in mice and marmosets (Krause et al., 2021; Mimura et al., 2021). However, the long-term chemogenetic action of DCZ remains unclear, specifically in terms of the duration of a single-dosage effect and the impact of repetitive doses over several days to weeks. These will provide knowledge sufficient for designing chronic chemogenetic experiments and, in addition, for developing potential clinical therapeutic applications.

Here we investigated the pharmacokinetics and *in vivo* occupancy of orally administered DCZ to determine a dose range suitable for DREADDs studies in monkeys. We then demonstrated that oral DCZ administration induced a chemogenetic behavioral effect lasting at least >4 h in macaque monkeys with hM4Di expressed in the prefrontal cortex (PFC). We also found that repetitive daily administrations of DCZ yielded consistent behavioral effects over weeks without apparent signs of desensitization.

## Materials and Methods

### Subjects

A total of 12 macaque monkeys [10 Japanese (*Macaca fuscata*) and 2 Rhesus monkeys (*Macaca mulatta*); 10 males, 2 females; 2.8-8.0 kg; age 4-10 years at the beginning of experiments] were used (Table 1). All experimental procedures involving animals were conducted in accordance with the Guide for the Care and Use of Nonhuman primates in Neuroscience Research (Japan Neuroscience Society; https://www.jnss.org/en/animal_primates) and were approved by the Animal Ethics Committee of the National Institutes for Quantum Science and Technology. The monkeys were kept in individual primate cages in an air-conditioned room (pair-housing environments are under preparation; see the preface of the above guideline). A standard diet, supplementary fruits/vegetables, and a tablet of vitamin C (200 mg) were provided daily.

**Table 1.** Summary of monkeys used in the study

ID	Species	Sex	Age (yr)	hM4Di	Occupancy	PK	DCZ uptake	Behavior
201	R	M	10	NA			✓	
221	J	M	7	NA			✓	
226	J	F	8	NA		✓		✓
228	R	M	7	NA			✓	
229	R	M	5	dlPFC			✓	✓
237	J	M	7	amygdala	✓			
239	J	M	6	NA				✓
240	J	M	6	NA			✓	
245	J	F	7	dlPFC		✓	✓	✓
248	J	M	4	NA		✓		
249	J	M	4	NA		✓		
253	J	M	5	NA				✓
Total	12 (R,2/J,10)	F,2; M,10		3	1	4	6	5

A tick indicates the subject was used in the experiment. PK, pharmacokinetics study; J, Japanese; R, Rhesus. #229 and #245 were used in our previous studies (Nagai et al., 2020; Oyama et al., 2021).

### Viral vector production

Adeno-associated virus (AAV) vector expressing hM4Di (AAV1-hSyn-hM4Di-IRES-AcGFP, 4.7 × 10^13^ particles/ml) was produced by helper-free triple transfection procedure, which was purified by affinity chromatography (GE Healthcare). Viral titer was determined by quantitative PCR using Taq-Man technology (Invitrogen).

### Surgical procedures and viral vector injections

Surgeries were performed under aseptic conditions in a fully equipped operating suite. We monitored body temperature, heart rate, SpO_2_, and tidal CO_2_ throughout all surgical procedures. Monkeys were immobilized by intramuscular (i.m.) injection of ketamine (5-10 mg/kg) and xylazine (0.2-0.5 mg/kg) and intubated with an endotracheal tube. Anesthesia was maintained with isoflurane (1%-3%, to effect). Before surgery, MRI (7 tesla 400 mm/SS system, NIRS/Kobelco/Brucker) and X-ray CT scans (Accuitomo170, J. Morita) were performed under anesthesia (continuous intravenous infusion of propofol 0.2-0.6 mg/kg/min). Overlay MR and CT images were created using PMOD image analysis software (PMOD Technologies) to estimate stereotaxic coordinates of target brain structures. After surgery, prophylactic antibiotics and analgesics (cefmetazole, 25-50 mg/kg/day; ketoprofen, 1-2 mg/kg/day; 7 d) were administered.

One monkey (#237) received injections of AAV vector into one side of the amygdala (2 μl × 2 locations). The monkey underwent a surgical procedure to open burr holes (∼8 mm diameter) for the injection needle. Viruses were pressure-injected using a 10 μl Hamilton syringe (model 1701 RN, Hamilton) with a 30-gauge injection needle and a fused silica capillary (Nagai et al., 2016). The Hamilton syringe was mounted into a motorized microinjector (UMP3T-2, WPI) that was held by a manipulator (model 1460, David Kopf) on the stereotaxic frame. After the dura mater was opened ∼3 mm, the injection needle was inserted into the brain and slowly moved down 2 mm beyond the target and then kept stationary for 5 min, after which it was pulled up to the target location. The injection speed was set at 0.25 μl/min. After each injection, the needle remained *in situ* for 15 min to minimize backflow along the needle.

Two monkeys (#229 and #245) received injections of AAV vector into the bilateral PFC (Brodmann's area 46) as described previously (Nagai et al., 2020; Oyama et al., 2021). The frontal cortex was exposed by removing a bone flap and reflecting the dura mater. Handheld injections at an oblique angle to the brain surface were made under visual guidance through an operating microscope (Leica M220, Leica Microsystems). Nine tracks were injected in each hemisphere located at the caudal tip (1 track) and along the dorsal (4 tracks) and ventral (4 tracks) bank of the principal sulcus. Viral vectors were injected at 3-5 μl per track depending on the depth. A total of 35-44 μl of viral aliquots was injected into each hemisphere.

### PET imaging

PET imaging was conducted as previously reported (Nagai et al., 2020). Briefly, PET scans were performed using a microPET Focus 220 scanner (Siemens Medical Solutions). Monkeys were immobilized by ketamine (5-10 mg/kg) and xylazine (0.2-0.5 mg/kg) and then maintained under anesthetized condition with isoflurane (1%-3%) during all PET procedures. Transmission scans were performed for ∼20 min with a Ge-68 source. Emission scans were acquired in 3D list mode with an energy window of 350-750 keV after intravenous bolus injection of [^11^C]DCZ (324.9-382.3 MBq). Emission data acquisition lasted for 90 min. To estimate the specific binding of [^11^C]DCZ, regional binding potential relative to nondisplaceable radioligand (BP_ND_) was calculated by PMOD with an original multilinear reference tissue model (MRTMo) (Yan et al., 2021). PET scans were conducted at 45 d after injection of vectors for the two monkeys (#229 and #245) and for one monkey expressing hM4Di in the amygdala (#237) between 496 and 984 d, during which enough hM4Di expression for the occupancy study was observed after its introduction.

### Drug administration

DCZ (MedChemExpress HY-42 110) was dissolved in 2.5% DMSO (Fujifilm Wako Pure Chemical), aliquoted, and stored at –30°C. For systemic intramuscular injection, this stock solution was diluted in saline to a final volume of 100 μg/kg, and injected intramuscularly. For systemic oral administration (p.o.), the DCZ stock solution was diluted in saline to a final volume of 100, 300, or 1000 μg/kg. The solution was then injected into some pieces of snack or diluted in drinking water, which were given to the monkeys. It typically took 2-3 min for the monkeys to consume. After each administration, we confirmed that there was no food in the monkeys' mouths. Fresh solution was prepared on each day of usage.

### Occupancy study

An occupancy study was performed using one monkey expressing hM4Di in the amygdala (#237), because it is ideal for placing voxels of interest and measuring tracer binding in a large volume of tissue expressing hM4Di and non-DREADD control on the other side, as in previous studies (Nagai et al., 2016, 2020). PET procedures were described above, except for pretreatment of DCZ or vehicle. Pretreatment of DCZ or vehicle p.o. via a gastric catheter was followed by anesthesia and emission scans at 30 and 90 min, respectively. The baseline scan data were obtained within 3 weeks from the day of PET scans for occupancy measurement to minimize the effect of hM4Di expression level changes during this period. To estimate the specific binding of [^11^C]DCZ, BP_ND_ was calculated by PMOD with MRTMo. The volume of interest for the hM4Di-positive region was defined as the area where the BP_ND_ value of [^11^C]DCZ was higher than 0.5, while that of the control region was placed at the corresponding contralateral side.

Estimates of fractional occupancy were calculated according to the previous study (Nagai et al., 2020) by the following equation:

Occ=(hM4DiBPBL−hM4DiBPPT)/(hM4DiBPBL−BCONPBL)

where *^hM4Di^BP_BL_* and *^CON^BP_BL_* indicate BP_ND_ at the hM_4_Di-expressing amygdala region and contralateral control region under baseline conditions, respectively, while *^hM4Di^BP_PT_* indicates BP_ND_ at the hM_4_Di-expressing amygdala region under pretreatment condition. The relationship between occupancy (*Occ*) and agonist dose (*D_DCZ_*) was modeled by the following Hill equation:

$$Occ=D_{DCZ}{}^{n}/(D_{DCZ}{}^{n}+ED_{50}{}^{n})$$

where *ED_50_* and *n* indicate the agonist dose achieving 50% occupancy and the Hill coefficient, respectively.

### Pharmacokinetics analysis

Three monkeys (#226, #248, and #249) were used to assess the concentration of DCZ and its metabolites in plasma or CSF. First, the monkeys were administered DCZ intramuscularly or orally (100 μg/kg for i.m.; 300 μg/kg for p.o.). Then they were immobilized by intramuscular injection of ketamine (5-10 mg/kg) and xylazine (0.2-0.5 mg/kg) and intubated with an endotracheal tube. Anesthesia was maintained with isoflurane (1%-3%, to effect). Then blood and CSF were collected at 30, 60, 90, 120, 150, 180, 210, and 240 min after DCZ administration; and on the next day, monkeys were again anesthetized, and the samples were collected at 24 h after DCZ administration. Blood was collected with a heparinized syringe, and plasma samples were obtained by centrifugation at 3500 × *g* for 15 min. All samples were stocked at −80°C until analysis.

The pretreatment protocols for CSF and plasma samples were described previously (Nagai et al., 2020). Quantification of DCZ, and its metabolites C21 and DCZ-N-oxide, was performed by multiple reaction monitoring using a Shimadzu UHPLC LC-30AD system (Shimadzu) coupled to a tandem MS AB Sciex Qtrap 6500 system (AB Sciex). The following multiple reaction monitoring transitions (Q1/Q3) were used to monitor each compound: DCZ (293.0/236.0), C21 (279.0/193.0), and DCZ-N-oxide (309.0/192.9). Other protocols were described previously.

A blood sample was also collected from one monkey (#245) following oral administration of 300 μg/kg of DCZ along with the daily behavioral testing under awake conditions to quantify the concentration of DCZ. The collected sample was treated and analyzed as well as those described above.

### Behavioral task

Three monkeys (#226, #229, and #245) were tested with a spatial delayed response task. The protocol was almost the same as in our previous study (Oyama et al., 2021). Behavioral testing was conducted in a sound-attenuated room. Monkeys sat on a monkey chair from which they could reach out one hand and take food to their mouths. A wooden table with two food-wells was placed in front of the monkeys, and a screen was placed between the monkeys and the table. First, a piece of food reward (raisin or snack) was placed in one of the two food-wells, and then both wells were covered with wooden plates. Then, the screen was placed for 0.5, 10, or 30 s, which served as delay periods. The position of the baited well (left or right) and the delay period were determined pseudo-randomly. After the delay period, the screen was removed and the monkeys were allowed to select either food-well. The monkeys were allowed to get the food if they reached for the correct food-well and removed the cover plate. The intertrial interval was set at 10 s. A daily session lasted ∼1 h, and consisted of three blocks of 30 trials for #229, and two blocks of 30 trials for #226 and #245, which were interleaved with 5 min rest periods. Behavioral tests began immediately or 0.5, 1, 3, or 24 h after vehicle or DCZ treatment.

Another monkey without AAV injection (non-DREADD; Monkey 239) was tested with a reward-size task using the same protocol as applied in a previous study (Minamimoto et al., 2009). The behavioral testing began 1 h after an oral administration of either vehicle or DCZ.

### Statistics

To examine the effect of each treatment on the performance of the delayed response task, behavioral measurement (correct rates) was subjected to Welch's *t* test or two-way ANOVA using GraphPad Prism 7.

### Histology and immunostaining

For histologic inspection, two monkeys (#229 and #245) were deeply anesthetized with an overdose of sodium pentobarbital (80 mg/kg, i.v.) and transcardially perfused with saline at 4°C, followed by 4% PFA in 0.1 m PBS, pH 7.4. The brain was removed from the skull, postfixed in the same fresh fixative overnight, saturated with 30% sucrose in PB at 4°C, and then cut serially into 50-μm-thick sections on a freezing microtome. For visualization of immunoreactive signals of GFP (coexpressed with hM4Di), a series of every sixth section was immersed in 1% skim milk for 1 h at room temperature and incubated overnight at 4°C with rabbit anti-GFP monoclonal antibody (1:200-500, G10362, Thermo Fisher Scientific) in PBS containing 0.1% Triton X-100 and 1% normal goat serum for 2 d at 4°C. The sections were then incubated in the same fresh medium containing biotinylated goat anti-rabbit IgG antibody (1:1000; Jackson ImmunoResearch Laboratories) for 2 h at room temperature, followed by avidin-biotin-peroxidase complex (ABC Elite, Vector Laboratories) for 2 h at room temperature. For visualization of the antigen, the sections were reacted in 0.05 m Tris-HCl buffer, pH 7.6, containing 0.04% DAB, 0.04% NiCl_2_, and 0.003% H_2_O_2_. The sections were mounted on gelatin-coated glass slides, air-dried, and cover-slipped. A part of other sections was Nissl-stained with 1% cresyl violet. Images of sections were digitally captured using an optical microscope equipped with a high-grade charge-coupled device camera (Biorevo, Keyence).

## Results

### Pharmacokinetics of orally administered DCZ

**Figure 1.**
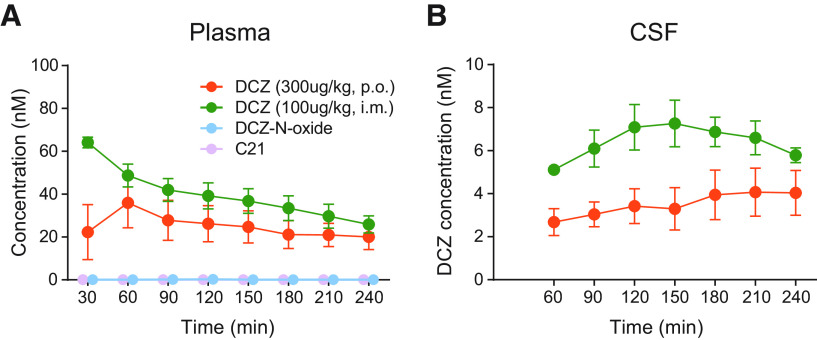
Time concentration profiles of DCZ by oral and intramuscular administration. ***A***, Time course of DCZ concentration in plasma (***A***) and CSF (***B***) following intramuscular injection of 100 μg/kg (green) and oral administration of 300 μg/kg (orange) DCZ. Concentration of major metabolites of DCZ (DCZ-N-oxide and C21, light blue and purple, respectively) following oral administration of 300 μg/kg DCZ is also shown (data for C21 were behind those of DCZ-N-oxide as neither was detected). Data were collected from three monkeys (#226, #248, and 249). Error bars indicate SEM.

We first conducted pharmacokinetic studies in three naive monkeys to estimate the time course of the chemogenetic effects of DCZ via oral administration. In our previous study, intramuscular administration of DCZ at 100 μg/kg provided a sufficient level of DCZ concentration in CSF to activate hM4Di receptors for 2 h, during which it gave rise to behavioral deficits in monkeys via hM4Di activation (Nagai et al., 2020). Given that oral administration of clozapine, an analog of DCZ, results in relatively lower bioavailability and slower onset of action compared with intravenous administration (Sun and Lau, 2000), we first examined DCZ concentrations in the plasma and CSF for 4 h following oral DCZ administration (300 μg/kg), as well as intramuscularly (100 μg/kg) as reference. Oral DCZ administration yielded a maximum DCZ concentration in plasma at 60 min after administration, followed by a gradual decrease (Fig. 1*A*, orange). By contrast, intramuscular administration increased plasma DCZ concentration at 30 min, which then decreased monotonically (Fig. 1*A*, green). Both oral and intramuscular administrations produced relatively stable DCZ concentrations in CSF for at least 4 h (>2 and >5 nM; Fig. 1*B*, orange and green, respectively), which were sufficient concentrations to activate hM4Di DREADD (EC_50_ of 0.081 nM) (Nagai et al., 2020). DCZ was not detected in plasma or CSF 24 h after administration. We also examined the concentration of possible metabolites of DCZ, C21, and DCZ-N-oxide in plasma; however, metabolites were not detected (Fig. 1*A*, light blue and purple), confirming the metabolic stability of DCZ (Nagai et al., 2020). Together, these results suggest that the oral administration of DCZ produces a relatively long-lasting pharmacokinetic profile without significant metabolite production and a lower bioavailability, ∼10%-20% of that of intramuscular administration.

### Measuring *in vivo* occupancy of hM4Di-DREADD by oral DCZ administration

**Figure 2.**
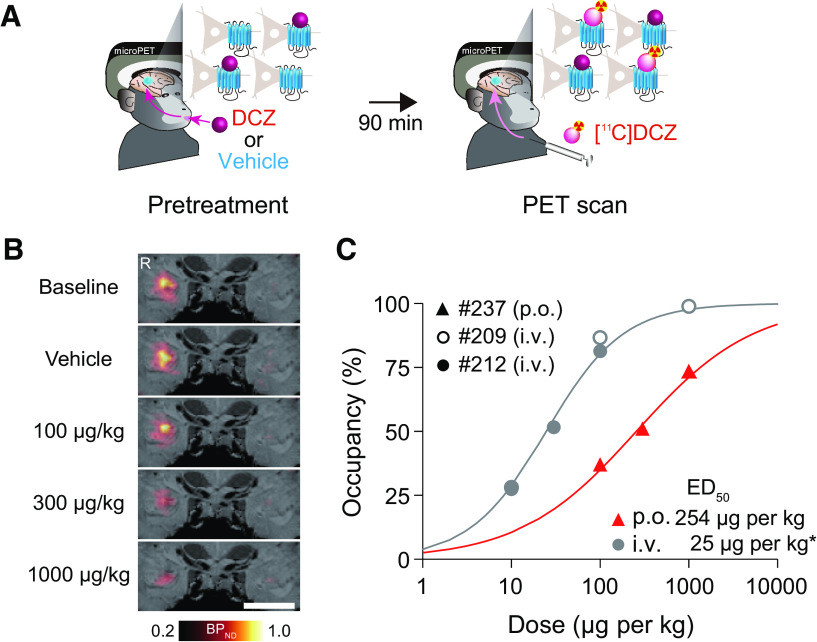
PET visualizes the occupancy of orally administered DCZ for hM4Di. ***A***, Schematic illustrations of occupancy study by PET. Monkeys underwent [^11^C]DCZ-PET scan 90 min after p.o. administration of nonradiolabeled DCZ or vehicle. ***B***, Coronal sections of parametric [^11^C]DCZ-PET image of specific binding (BP_ND_) overlaying MR image of a monkey expressing hM4Di in the amygdala. Scale bars, 10 mm. ***C***, Occupancy of hM4Di plotted as a function of DCZ. Red triangles represent occupancy by oral DCZ doses obtained from Monkey 237. Curves are the best-fit Hill equation for the data. ED_50_ indicates the agonist dose inducing 50% occupancy. Hill coefficient (*n*) and coefficient of determination (*R*^2^) values are as follows: p.o.: *n* = 0.66, *R*^2^ = 0.988; i.v.: *n* = 1, *R*^2^ = 0.99. The data for intravenous injection (gray) refer to a previous study (Nagai et al., 2020).

Next, to determine an effective and safe dose range for oral DCZ administration, we measured hM4Di receptor occupancy by DCZ: the relationship between DCZ dose and the degree of hM4Di receptor occupation (Fig. 2*A*). This was important because a higher agonist dose will afford a stronger chemogenetic effect but may risk higher off-target effects (Upright and Baxter, 2020). Normally, proportional receptor occupancy is a generalizable measure because it reflects the relationship between target drug concentration and its receptor affinity, regardless of brain region or receptor density (Cunningham et al., 2010; Takano et al., 2016). In our previous study, a series of PET studies was used to determine the optimal DCZ dose that would yield a 50%-80% occupancy of hM4Di, which was 30-100 μg/kg for systemic administration (Fig. 2*C*) (for details, see Nagai et al., 2020). Given that the bioavailability of oral administration would be 10%-20% of that of systemic administration (Fig. 1), we estimated that oral DCZ doses achieving 50% and 80% hM4Di occupancy would be ∼300 and 1000 μg/kg, respectively. We performed a PET occupancy study using a monkey that had received injection of a viral vector encoding an hM4Di gene into the unilateral amygdala (see Materials and Methods).

Baseline PET imaging with radiolabeled DCZ ([^11^C]-DCZ) confirmed that the hM4Di expression at the injected side of the amygdala was indicated by an increased radioligand binding (Fig. 2*B*, baseline). Subsequent [^11^C]-DCZ PET scans were performed after oral pretreatments (i.e., oral administration of vehicle, 100, 300, or 1000 μg/kg of nonradiolabeled DCZ). Consistent with our previous findings, specific binding of [^11^C]DCZ at the hM4Di-expressing amygdala region was diminished with increasing doses of DCZ (Fig. 2*B*). Occupancy was determined as a reduction of specific radioligand binding (BP_ND_) at the target region over the control side relative to baseline (Fig. 2*B*; for details, see Materials and Methods). As we predicted, the hM4Di occupancies with 100, 300, and 1000 μg/kg oral administration were ∼35%, 50%, and 70%, respectively (Fig. 2*C*). The dose for 50% occupancy (EC_50_) was 254 µg/kg, which corresponded to an ∼10-fold dose of systemic administration and was roughly consistent with the bioavailable profile shown in our pharmacokinetic study described above. Based on these results in pharmacokinetic and occupancy studies, we determined the effective and safety dose range for oral DCZ administration to be 300-1000 μg/kg.

### Oral DCZ administration selectively induces longer-lasting behavioral effects in hM4Di-expressing monkeys

**Figure 3.**
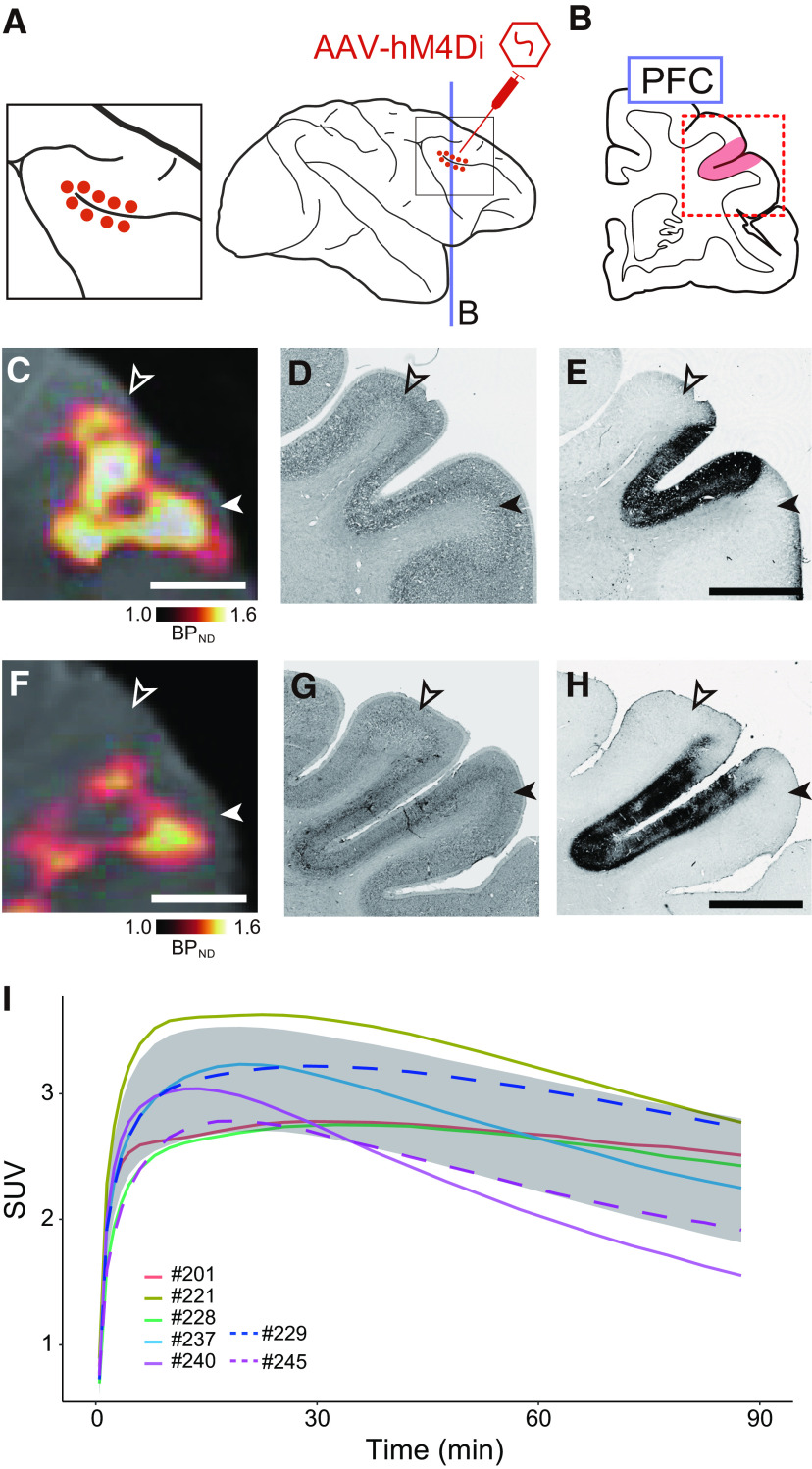
Introduction of hM4Di in bilateral dlPFC. ***A***, Illustration showing the injection sites for AAV vector carrying an hM4Di gene. Left, Zoomed-in view of the framed area of the right panel. ***B***, A coronal plane corresponding to the lines in the lateral view. Red dots and shaded area represent intended injection sites. ***C***, ***F***, *In vivo* visualization of hM4Di expression in dlPFC for #229 (***C***) and #245 (***F***). Coronal PET image showing specific binding of [^11^C]DCZ overlaying MR images. ***D***, ***G***, Corresponding Nissl-stained sections for #229 (***D***) and #245 (***G***). ***E***, ***H***, Corresponding DAB-stained sections representing immunoreactivity against a reporter protein for #229 (***E***) and #245 (***H***). Open and filled arrowheads represent the dorsal and ventral borders of the target regions, respectively. Scale bars, 5 mm. ***I***, The time course of whole-brain uptake of [^11^C]DCZ for seven monkeys, including #229 and #245. Shaded area represents the SD of five monkeys, excluding #229 and #245. #229 and #245 were the same subjects as used in our previous study (Oyama et al., 2021). SUV, Standardized uptake value [regional radioactivity (Bq cm^−3^) × body weight (g)/injected radioactivity (Bq)].

We next examined the efficacy and time course of the chemogenetic effect of oral DCZ administration using hM4Di-expressing monkeys. We used two monkeys with hM4Di expressed in the bilateral dlPFC around the principal sulcus (Brodmann's area 46; Fig. 3*A*,*B*), a brain region known to be responsible for spatial working memory and executive function (Fuster, 2015). Several weeks after the injections, we performed PET imaging with [^11^C]DCZ to visualize hM4Di expression noninvasively. High levels of [^11^C]DCZ binding (BP_ND_ > 1.0) were localized in the bilateral dlPFC in both monkeys, specifically from the caudal end of the principal sulcus to 5-6 mm anterior to it (Fig. 3*C*,*F*). These PET signals reflected hM4Di expression, as postmortem analysis confirmed GFP-positive zone in dorsal and ventral bank of the principal sulcus (Fig. 3*D–H*). When we compared the time course of [^11^C]DCZ uptake in the whole brain of these monkeys with that of other monkeys that had not undergone surgery (Fig. 3*I*), there was no marked difference across the subjects. This suggested that the brain permeability of DCZ was unaffected by the dlPFC surgery.

**Figure 4.**
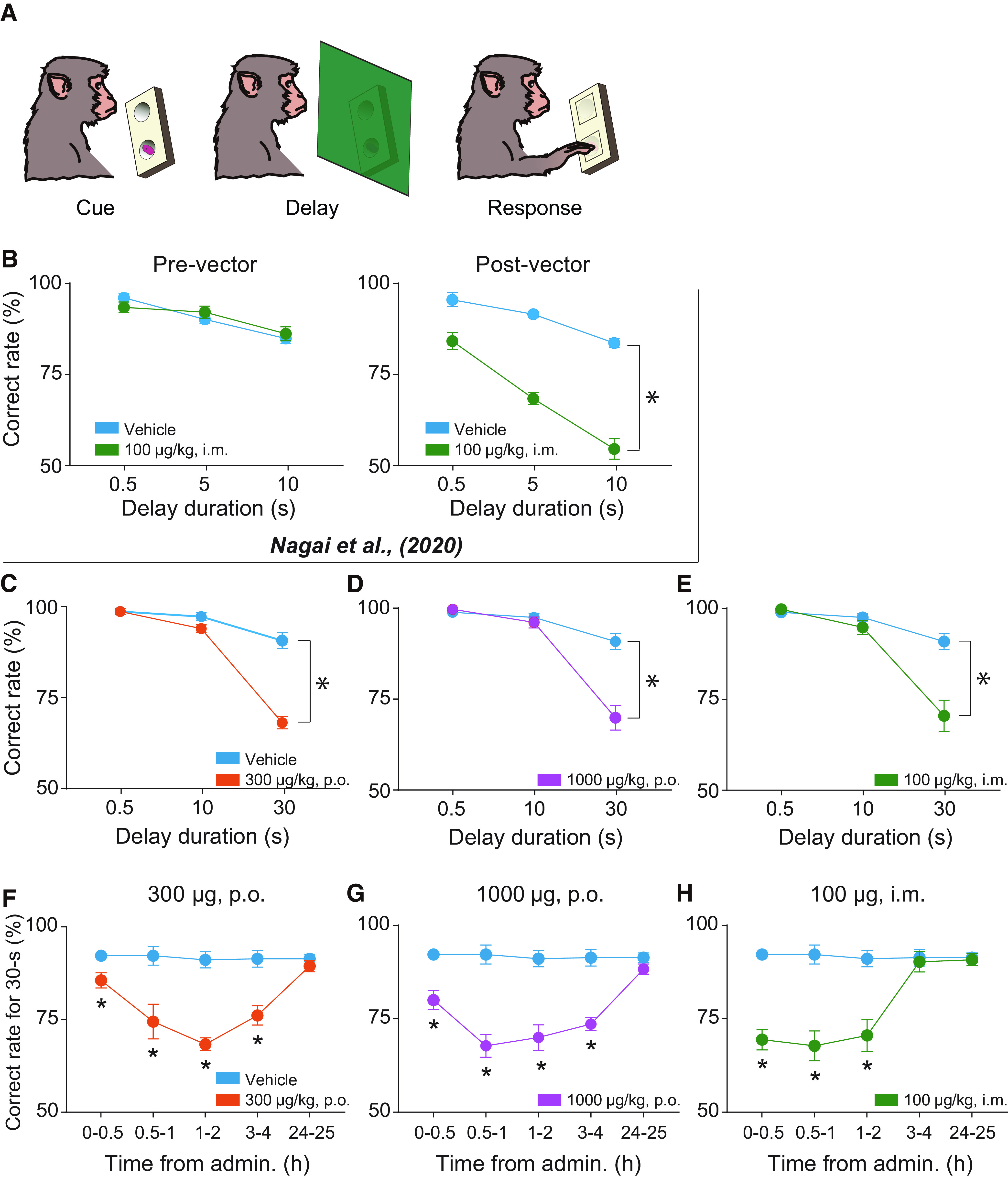
Effects of oral and intramuscular administration of DCZ on a cognitive task in monkeys expressing hM4Di in the dlPFC. ***A***, Delayed response task. ***B***, Effects of intramuscular administration of 100 μg/kg of DCZ on task performance for #245 before (Pre-vector) and after (Post-vector) introduction of hM4Di (Nagai et al., 2020). Two-way ANOVA (treatment × delay) revealed significant main effects of treatment after introduction of hM4Di (treatment; *F*_(1,24)_ = 188, *p* = 7.3 × 10^−13^; delay; *F*_(2,24)_ = 57.3, *p* = 7.3 × 10^−10^; interaction; *F*_(2,24)_ = 10.9, *p* = 4.3 × 10^−4^), but not before its introduction (treatment; *F*_(1,24)_ = 0.03, *p* = 0.86; delay; *F*_(2,24)_ = 19.9, *p* = 8.0 × 10^−7^; interaction; *F*_(2,24)_ = 1.4, *p* = 0.26). ***C-E***, Effects of oral administration of 300 μg/kg (orange) and 1000 μg/kg (magenta), and intramuscular administration of 100 μg/kg (green) of DCZ on task performance. The same data for the vehicle were plotted in each panel (cyan). The behavioral tests were conducted 1-2 h following each administration. In all conditions, two-way ANOVA (treatment × delay) revealed significant main effects of treatment and delay, and interaction (100 μg/kg, i.m.; treatment: *F*_(1,30)_ = 17.4, *p* = 2.3 × 10^−4^; delay: *F*_(2,30)_ = 41.6, *p* = 2.2 × 10^−9^; interaction: *F*_(2,30)_ = 13.5, *p* = 6.4 × 10^−5^; 300 μg/kg, p.o.; treatment; *F*_(1,30)_ = 60.9, *p* = 1.1 × 10^−8^; delay; *F*_(2,30)_ = 117.1, *p* = 7.0 × 10^−14^; interaction; *F*_(2,30)_ = 40.5, *p* = 3.0 × 10^−9^; 1000 μg/kg, p.o.; treatment; *F*_(1,30)_ = 22.9, *p* = 4.2 × 10^−5^; delay; *F*_(2,30)_ = 62.7, *p* = 1.9 × 10^−11^; interaction; *F*_(2,30)_ = 21.4, *p* = 1.7 × 10^−6^), indicating the delay-dependent behavioral deficits. The data from two monkeys were pooled (*N* = 6 sessions; three sessions for each monkey). ***F-H***, Time course of behavioral effects of each administration. Only the correct rates of 30 s trials were plotted, as they were most prominent, as shown in ***C-E***. *Significant differences compared with vehicle administrations (*p* < 0.05, Welch's *t* test, uncorrected). Error bars indicate SEM.

Our previous studies have shown that chemogenetic silencing of the dlPFC via intramuscular DCZ administration (100 μg/kg) in these monkeys impaired performance in the spatial delayed response task (Fig. 4*A*) (Nagai et al., 2020; Oyama et al., 2021). Before the introduction of hM4Di, intramuscular DCZ administration (100 μg/kg) per se did not have an impact on working memory in #245 (Fig. 4*B*) (Nagai et al., 2020), suggesting that oral administration of DCZ <1000 μg/kg would be expected not to have any side effects. Then we directly compared the effects of oral administration with those of intramuscular injections by using the same subjects as in our previous studies that showed similar behavioral deficits (Nagai et al., 2020), which would enable us to demonstrate that oral DCZ administration can be adapted to dlPFC, one of the core brain regions specialized for primates. We examined the behavioral effects of two oral DCZ doses (300 and 1000 μg/kg) and an intramuscular DCZ dose (100 μg/kg) for comparison. Consistent with previous studies (Nagai et al., 2020; Oyama et al., 2021), intramuscular 100 μg/kg DCZ administration resulted in a delay-dependent decrease in the task performance for 1-2 h following administration (Fig. 4*E*). Oral DCZ administration of 300 and 1000 μg/kg DCZ also induced a comparable impairment of the task performance for 1-2 h after administration (Fig. 4*C*,*D*). We further assessed the time course of the chemogenetic effects by conducting a behavioral examination at 5 different time windows after treatment: 0-0.5, 0.5-1, 1-2, 3-4, and 24-25 h. We focused on the performance of the trials with the longest delay period, where the effects were most prominent (Fig. 4*C–E*, 30 s). In the first 30 min period, intramuscular administration significantly decreased the correct rates (Fig. 4*H*, green) compared with those after vehicle administration (Fig. 4*H*, cyan), indicating rapid behavioral effects as previously demonstrated (Nagai et al., 2020). Significant effects were also observed following oral administration of 300 and 1000 μg/kg (Fig. 4*F*,*G*, orange and magenta, respectively). Similarly, all treatments had significant effects at 0.5-1 and 1-2 h after administration (Fig. 4*F–H*). At 3-4 h after administration, intramuscular administration had no influence on performance (Fig. 4*H*), while the oral administrations remained effective (Fig. 4*F*,*G*).

To determine the minimum effective dose of DCZ via oral administration, we also administered 100 μg/kg DCZ orally and examined the monkeys 1-2 and 3-4 h thereafter. The performance was significantly impaired after 1-2 h (Welch's *t* test, *t* = 4.2, *p* = 0.002), but not after 3-4 h (*t* = 0.48, *p* = 0.65), indicating that this dose is effective only for a shorter duration compared with 300 and 1000 μg/kg.

**Figure 5.**
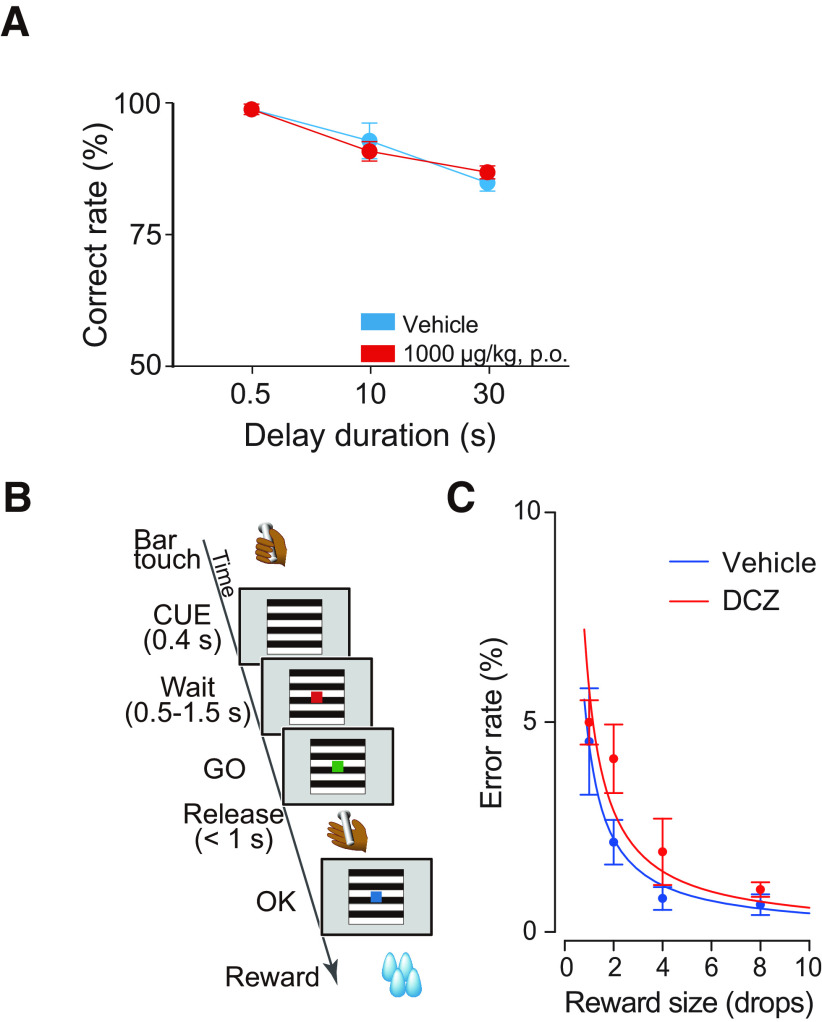
Effects of 1000 µg/kg DCZ oral administration on behavioral tasks in non-DREADD animals. ***A***, Performance of delayed response task following oral administration of vehicle (cyan) and 1000 μg/kg DCZ (red) in a non-DREADD animal (#226). There was no significant difference between treatments (two-way ANOVA, treatment × delay, treatment: *F*_(1,24)_ = 0, *p* = 1). ***B***, Illustration of a reward-size task. Each trial in this task began when the monkey touched a lever, which was followed by the appearance of a visual cue signaling the size of the upcoming reward (1, 2, 4, or 8 drops). To obtain the reward, the monkeys had to release the lever when a visual target changed color from red to green. ***C***, Example behavioral data of reward size task for DCZ (1000 µg/kg, p.o.; red) and vehicle administration session (cyan) obtained from a non-DREADD monkey (#239). Two-way ANOVA (treatment × reward size) revealed no significant difference (*p* > 0.05). Error bars indicate SEM.

Importantly, the decrease in task performance was found to be dependent on delay duration (Fig. 4*C*), suggesting that the orally administered DCZ resulted in loss of dlPFC function, but did not produce side effects on other functions, such as general attention or motivation. To further confirm that oral administration had no unwanted side effects, we conducted control experiments with the delayed response task (Fig. 5*A*) and a simple instrumental task (Fig. 5*B*,*C*) in two monkeys to whom DREADDs had not been introduced (non-DREADD monkeys). Similar to our previous study that tested with 100 μg/kg of intramuscular DCZ administration (Nagai et al., 2020), oral DCZ administration of 1000 μg/kg did not produce any discernible behavioral changes in either task (Fig. 5*A*,*C*), suggesting that oral DCZ administration itself is behaviorally inert at a dose of ≤1000 μg/kg.

Together, these results suggest that oral DCZ administration can induce behavioral effects selectively in hM4Di-expressing monkeys for hours without adverse side effects; specifically, a dose of 300-1000 µg/kg is effective for at least 4 h.

### Chronic DCZ administration enables successive induction of chemogenetic silencing for weeks

**Figure 6.**
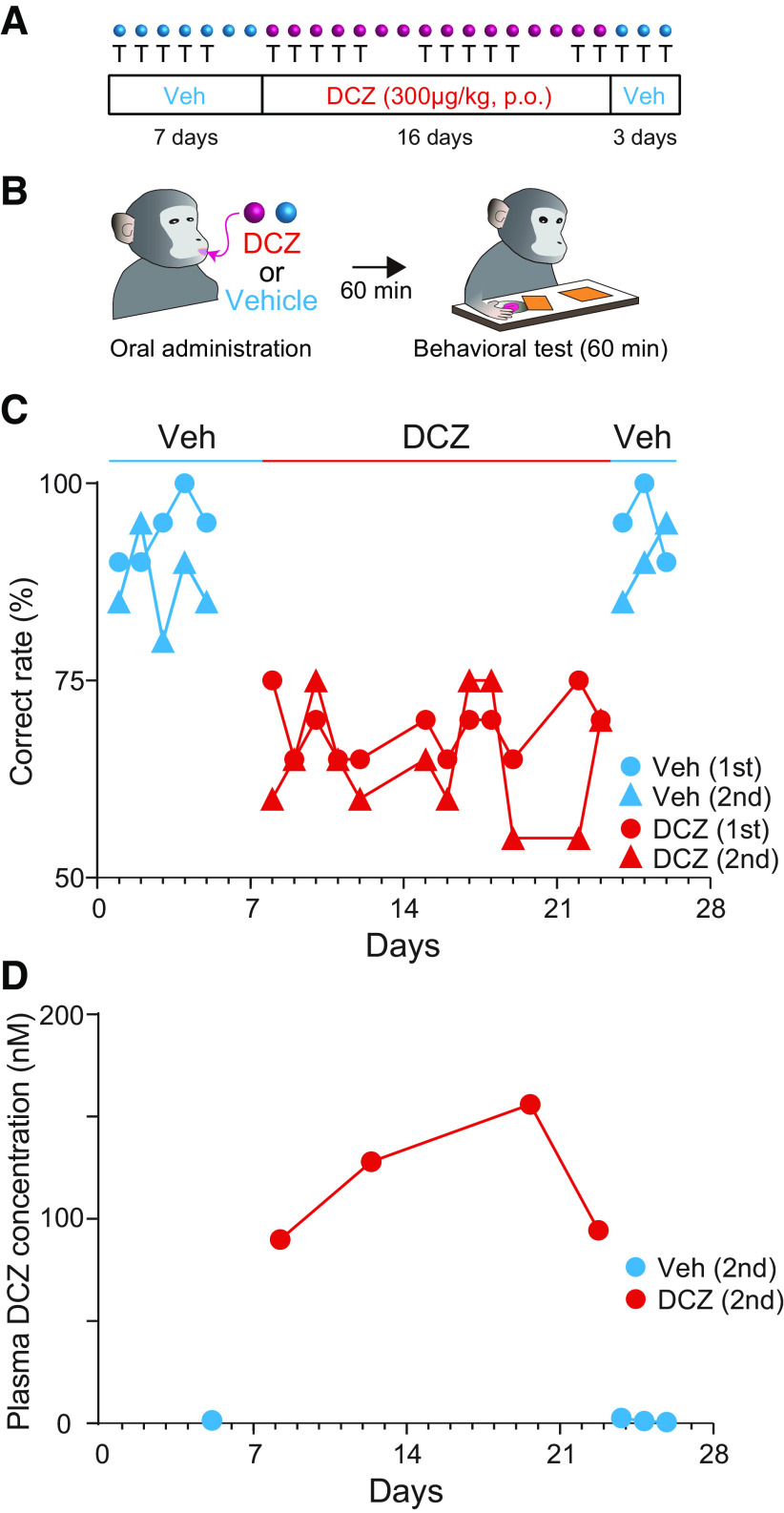
Chronic behavioral effect of daily DCZ administration across weeks. ***A***, Schedule of experiments. In a series of sessions, after 7 d of vehicle administrations (Veh), DCZ was orally administered for 16 d, followed by 3 d of vehicle, once each day. The monkey (#245) was tested by delayed response task over 5, 12, and 3 d, respectively. The series of testing were repeated twice. Cyan and purple circles represent the drugs administered (vehicle or DCZ). “T” below the circles indicates the days on which the behavioral tests were conducted. ***B***, Schedule of an experiment in one day. ***C***, Correct rates plotted as a function of days for vehicle (cyan) and DCZ (red) administrations for the first (circle) and second (triangle) series, respectively. There was significant difference between vehicle and DCZ administrations (two-way repeated measures ANOVA with treatment and schedule [first and second schedules], main effect of treatment, *F*_(1,18)_ = 194, *p* = 4.4 × 10^−11^). There was no significant difference between performance during the former and latter periods (two-way repeated measures ANOVA with timing [former vs latter] and schedule, main effect of timing, *F*_(1,10)_ = 0.03, *p* = 0.87), indicating that the impairment was consistent during the period of administration. ***D***, Plasma DCZ concentration plotted as a function of days for vehicle (cyan) and DCZ (red) administrations for the same monkey (#245). Data were collected as with the behavioral data (***D***), that is, 1 h after oral administration of 300 μg/kg DCZ. The DCZ concentration appeared to be relatively higher than those of other monkeys in Figure 1*A*. This might be attributable to the difference of sampling conditions (i.e., under anesthesia or awake conditions; for details, see Materials and Methods). It is also possible that oral administration of DCZ may be less efficient for crossing the blood–brain barrier in animal #245, since the PET data suggest that brain uptake of DCZ in #245 was relatively low compared with the other animals (although it was still within a standard range; Fig. 3*I*).

Finally, we investigated the efficacy of chronic DCZ administration across weeks. One monkey expressing hM4Di in the dlPFC was tested daily with the delayed response task to examine the chemogenetic effect of repetitive oral DCZ administration. Chronic oral DCZ administrations for at least two weeks were preceded and followed by several days of control vehicle administration (Fig. 6*A*,*B*), and repeated twice with a sufficient interval of several months. This design allowed us to investigate whether repetitive DCZ dose would consistently induce behavioral deficits for weeks and how soon task performance would be recovered after withdrawal of DCZ. Daily oral DCZ administrations (300 µg/kg) consistently induced impairment of the performance of the delayed response task during 1-2 h after administration compared with that of following vehicle injections throughout the repetitive oral DCZ period (Fig. 6*C*), indicating that apparent desensitization did not occur. Previous studies have shown that chronic chemogenetic manipulation caused post-treatment effects, such as rebound excitability (Desloovere et al., 2019; Goossens et al., 2021), suggesting that it could induce some physiological changes lasting even after agonist withdrawal. That was not the case in the current study: we found that performance recovered to a normal level on the first day after withdrawing from chronic DCZ administration (Fig. 6*C*), indicating that successive behavioral deficits were the outcome of temporal chemogenetic silencing rather than because of physiological change, such as DREADD-induced plasticity.

It has been shown that plasma concentrations of the DCZ analog clozapine vary within subjects over weeks of treatment (Centorrino et al., 1994), especially dropping by an average of >50% in the first 12 weeks (Kurz et al., 1998). We confirmed that the concentration of DCZ following daily administration was maintained at a similar level throughout the period of DCZ administration, indicating that chronic manipulation did not alter the pharmacokinetics of DCZ for at least two weeks (Fig. 6*D*). We also verified that chronic DCZ administration for a single schedule (16 d for DCZ, and 7 and 3 d of vehicle administration; see Fig. 6*A*) alone had no significant impact on spontaneous movements in a non-DREADD monkey (#253; Welch's *t* test, *t* = 0.55, *p* = 0.59). Together, these results suggested that DCZ is suitable for a chronic and reversible manipulation of neuronal activity, leading to potential therapeutic applications in the future.

## Discussion

Here we demonstrated that oral administration of DCZ is an effective and noninvasive means for manipulating neuronal activity through hM4Di receptors in macaque monkeys. Our pharmacokinetics and *in vivo* PET occupancy examination showed that the bioavailability of orally administered DCZ was ∼10%-20% of that of intramuscular injection; thus, the appropriate dosage of oral DCZ to exert hM4Di-mediated chemogenetic effects was estimated to be 300-1000 μg/kg. Indeed, this oral DCZ at this range of doses caused severe impairments in working memory performance for several hours in monkeys with hM4Di expressed in dlPFC but did not induce any discernible behavioral side effects in control non–DREADD-expressing monkeys. Furthermore, repetitive daily oral DCZ doses over two weeks yielded consistent chemogenetic effects without apparent signs of desensitization. Together, the oral administration of DCZ affords a minimally invasive strategy for chronic and reversible chemogenetic control of neuronal activity via muscarinic DREADDs in nonhuman primates, thereby providing a great potential for its clinical application.

For DREADDs studies in rodents and nonhuman primates, systemic (intraperitoneal, intramuscular, or intravenous) injections are the standard approach of agonist administration, as they allow precise control of dosage and timing. However, such approach forces the animals to be restrained, which may cause stress responses and unwanted effects on behavioral actions. Several DREADDs studies have demonstrated that oral administration seems effective for overcoming this issue. For example, oral CNO administration induced changes in ethanol consumption by inhibition of the nucleus accumbens via hM4Di, in which neuronal activity was remotely manipulated while the animals were unrestrained (Cassataro et al., 2014). This is beneficial when wanting to examine the effect on natural behavior, including social communication (Zou et al., 2016). Oral CNO administration has also been applied to manipulate the activity of non-neuronal cells, such as microglia (Grace et al., 2016). However, recent reports have revealed that the effects of CNO are partly mediated by its back-conversion to clozapine, which crosses the blood–brain barrier and acts as a DREADDs actuator (Gomez et al., 2017). Oral administration of low doses of clozapine and olanzapine as agonists has also been attempted (Goossens et al., 2021). Because of their high affinities for endogenous receptors, however, possible side effects are always a matter of concern that cannot completely be ruled out.

In the current study, we evaded these issues by using DCZ, a novel DREADDs agonist with high brain permeability and high selectivity. Since the effects of drugs on the CNS depend on multiple factors, such as brain permeability, drug kinetics, and affinity for target receptors, it is generally difficult to predict the optimal drug dosage. As we have demonstrated in previous studies (Nagai et al., 2016, 2020), PET occupancy measurements together with pharmacokinetics analysis are extremely effective ways for seeking the adequate agonist dose range. We found that the bioavailability of orally administered DCZ was 10%-20% compared with systemic injections (Figs. 1 and 2). Based on this estimation, the dose range suitable for hM4Di activation was determined to be 300-1000 μg/kg. In line with the kinetics data, such oral DCZ doses induced relatively long-lasting effects compared with intramuscular injections. The DCZ kinetics and behavioral effects were examined up to 4 h after oral administration, during which the concentration of DCZ persisted in CSF; thus, the chemogenetic effects may be maintained longer.

The working-memory impairment following DCZ administration cannot be explained by side effects because of action on endogenous receptors as indicated by other prior DREADDs agonists for the following reasons. First, one of the two monkeys (#245) had been tested with the delayed response task before introducing hM4Di in the dlPFC, showing that DCZ alone had no impact on the behavior (Fig. 4*B*). Second, control experiments using non–DREADD-expressing monkeys confirmed that these oral DCZ doses alone did not produce any discernible changes in behavior associated with spatial working memory or reward expectation (Fig. 5). Third, PET and histologic data suggest that two monkeys consistently express hM4Di, covering the similar region of dlPFC. Collectively, we concluded that behavioral results consistently observed in these two monkeys were because of the inactivation of dlPFC neurons via hM4Di activation.

In the present study, we did not examine the effects of oral DCZ administration on hM3Dq, an excitatory muscarinic DREADDs that responds to DCZ (Nagai et al., 2020). Our previous work showed that the lower doses (1-3 μg/kg) of DCZ through intramuscular injection were capable of inducing significant neuronal excitation in mice and monkeys expressing hM3Dq (Nagai et al., 2020). Thus, 3-30 μg/kg would be an effective range for activating hM3Dq via oral administration. Indeed, we have recently demonstrated that marmosets expressing hM3Dq produced consistent behavioral changes when fed a diet containing 10 μg/kg of DCZ (Mimura et al., 2021). In any case, it should be noted that the optimal agonist dose generally depends on the level of the overexpressed functional protein (Upright et al., 2018) and the targeted circuit to be manipulated. In addition, as is usual in studies using genetic methods, control experiments (e.g., using non-DREADDs animals) are recommended.

It has been shown that chronic administration of DREADDs agonists induces DREADDs-mediated changes for days to weeks in rodents (Nation et al., 2016; Desloovere et al., 2019; Paretkar and Dimitrov, 2019; Goossens et al., 2021). However, some studies reported that the effects following administrations of agonists were not consistent during treatments. For example, Goossens et al. (2021) reported that the administrations of clozapine and olanzapine caused significant behavioral changes for several days, but that such effects subsequently diminished, although the agonist administrations continued. In addition, it has been reported that inhibition by hM4Di with agonist treatment over weeks induced post-treatment rebound excitability (Desloovere et al., 2019; Goossens et al., 2021). In contrast to these studies, we showed that daily oral DCZ delivery constantly impaired the monkey's performance throughout the administration periods (Fig. 6). Moreover, performance on the day following completion of repeated doses of DCZ was as high as the baseline, suggesting that weeks-long inactivation of the PFC did not affect subsequent behavior. These results suggest that chronic DCZ administration did not cause desensitization of hM4Di or any long-lasting change, such as plasticity of neural circuits that govern working memory. It remains to be clarified whether this is a general capability of DREADD/DCZ or is because of the characteristics of neurons and local circuits in the dlPFC as a target. Irrespective of the mechanism, the present results indicate that it is possible to investigate the effects of chronic attenuation of PFC activity on various cognitive functions. Importantly, it can resolve potential discrepancies that may arise in the behavioral outcomes of different durations or methods of inactivation. For example, it has been demonstrated that acute and chronic silencing of PFC interneurons had opposite impacts on anxiety-like behavior in mice (Soumier and Sibille, 2014), suggesting that short- versus long-term manipulations of local circuits may differentially alter PFC network functions. In addition, long-term abnormalities or imbalances in prefrontal activity have been suggested as a pathophysiological mechanism for psychiatric disorders, since chronic stress exposure could lead to anxiety disorder and depression (Zhou et al., 2020). DREADDs subserve to mimic or reverse such long-term circuitry changes that are beyond the reach of conventional acute blockade and/or irreversible lesioning. The chemogenetic approach using DCZ as an actuator introduced in the present study will expand the opportunity of acute and chronic manipulations of specific circuits in the primate PFC, thereby leading to a better understanding of higher brain functions (e.g., working memory, decision-making, and action-planning, in health and disease conditions). This can be done without causing pain/stress to monkeys, and thus is also suitable for quantifying the effects on unrestrained naturalistic, social behavior (e.g., using a markerless motion tracking system) (Bala et al., 2020; Labuguen et al., 2021). In addition, it has the potential to be used in therapeutic applications for symptoms caused by abnormal neuronal activity in a specific brain region, such as seizures in epilepsy.

Finally, it should be noted that this study did not examine continuous chemogenetic effects beyond the duration of action of a single oral dose (∼4 h). Continuous silencing may be achieved with more frequent and repeated administrations. It should also be noted that the utility of chronic oral administration and the effect of DCZ in pre-DREADD condition were examined in a single monkey (#245). Future studies will need to determine the dosage and interval of administrations to maintain sufficient DCZ concentration to attain continuous chemogenetic effects, and to ensure that it does not cause undesirable side effects, toxicity, or tachyphylaxis, with appropriate within-subject control using non-DREADD animals.

DREADDs technology is becoming increasingly popular as a means of controlling neuronal activity remotely, less invasively and reproducibly in rodents and nonhuman primates. With the accumulation of numerous successful reports, muscarinic DREADDs are now under consideration for clinical application. Given the long-term stable chemogenetic effects with orally delivered DCZ in macaque monkeys, it holds great promise for the translational use of DREADDs technology, especially in the development of therapeutic trials for neurologic and neuropsychiatric disorders.

## References

[B1] Andreoli M, Marketkar T, Dimitrov E (2017) Contribution of amygdala CRF neurons to chronic pain. Exp Neurol 298:1–12. 10.1016/j.expneurol.2017.08.010 28830762 PMC5658242

[B2] Bala PC, Eisenreich BR, Yoo SB, Hayden BY, Park HS, Zimmermann J (2020) Automated markerless pose estimation in freely moving macaques with OpenMonkeyStudio. Nat Commun 11:4560. 10.1038/s41467-020-18441-5 32917899 PMC7486906

[B3] Bonaventura J, Eldridge MA, Hu F, Gomez JL, Sanchez-Soto M, Abramyan AM, Lam S, Boehm MA, Ruiz C, Farrell MR, Moreno A, Galal Faress IM, Andersen N, Lin JY, Moaddel R, Morris PJ, Shi L, Sibley DR, Mahler SV, Nabavi S, et al. (2019) High-potency ligands for DREADD imaging and activation in rodents and monkeys. Nat Commun 10:4627. 10.1038/s41467-019-12236-z 31604917 PMC6788984

[B4] Cassataro D, Bergfeldt D, Malekian C, Van Snellenberg JX, Thanos PK, Fishell G, Sjulson L (2014) Reverse pharmacogenetic modulation of the nucleus accumbens reduces ethanol consumption in a limited access paradigm. Neuropsychopharmacology 39:283–290. 10.1038/npp.2013.184 23903031 PMC3870771

[B5] Centorrino F, Baldessarini RJ, Kando JC, Frankenburg FR, Volpicelli SA, Flood JG (1994) Clozapine and metabolites: concentrations in serum and clinical findings during treatment of chronically psychotic patients. J Clin Psychopharmacol 14:119–125. 8195452

[B6] Cunningham VJ, Rabiner EA, Slifstein M, Laruelle M, Gunn RN (2010) Measuring drug occupancy in the absence of a reference region: the Lassen plot re-visited. J Cereb Blood Flow Metab 30:46–50. 10.1038/jcbfm.2009.190 19738632 PMC2949110

[B7] Desloovere J, Boon P, Larsen LE, Merckx C, Goossens MG, Van den Haute C, Baekelandt V, De Bundel D, Carrette E, Delbeke J, Meurs A, Vonck K, Wadman W, Raedt R (2019) Long-term chemogenetic suppression of spontaneous seizures in a mouse model for temporal lobe epilepsy. Epilepsia 60:2314–2324. 10.1111/epi.16368 31608439

[B8] Fuster JM (2015) The prefrontal cortex. Boston: Academic.

[B9] Gomez JL, Bonaventura J, Lesniak W, Mathews WB, Sysa-Shah P, Rodriguez LA, Ellis RJ, Richie CT, Harvey BK, Dannals RF, Pomper MG, Bonci A, Michaelides M (2017) Chemogenetics revealed: DREADD occupancy and activation via converted clozapine. Science 357:503–507. 10.1126/science.aan2475 28774929 PMC7309169

[B10] Goossens M, Boon P, Wadman W, van den Haute C, Baekelandt V, Verstraete AG, Vonck K, Larsen LE, Sprengers M, Carrette E, Desloovere J, Meurs A, Delbeke J, Vanhove C, Raedt R (2021) Long-term chemogenetic suppression of seizures in a multifocal rat model of temporal lobe epilepsy. Epilepsia 62:659–670. 10.1111/epi.16840 33570167

[B11] Grace PM, Strand KA, Galer EL, Urban DJ, Wang X, Baratta MV, Fabisiak TJ, Anderson ND, Cheng K, Greene LI, Berkelhammer D, Zhang Y, Ellis AL, Yin HH, Campeau S, Rice KC, Roth BL, Maier SF, Watkins LR (2016) Morphine paradoxically prolongs neuropathic pain in rats by amplifying spinal NLRP3 inflammasome activation. Proc Natl Acad Sci USA 113:E3441–E3450. 10.1073/pnas.1602070113 27247388 PMC4914184

[B12] Hirabayashi T, Nagai Y, Hori Y, Inoue K, Aoki I, Takada M, Suhara T, Higuchi M, Minamimoto T (2021) Chemogenetic sensory fMRI reveals behaviorally relevant bidirectional changes in primate somatosensory network. Neuron 109:3312–3322.e5. 10.1016/j.neuron.2021.08.032 34672984

[B13] Hori Y, Mimura K, Nagai Y, Fujimoto A, Oyama K, Kikuchi E, Inoue K, Takada M, Suhara T, Richmond BJ, Minamimoto T (2021) Single caudate neurons encode temporally discounted value for formulating motivation for action. eLife 10:e61248. 10.7554/eLife.61248 34328413 PMC8352586

[B14] Karbwang J, Na-Bangchang K, Congpuong K, Molunto P, Thanavibul A (1997) Pharmacokinetics and bioavailability of oral and intramuscular artemether. Eur J Clin Pharmacol 52:307–310. 10.1007/s002280050295 9248770

[B15] Krause WC, Rodriguez R, Gegenhuber B, Matharu N, Rodriguez AN, Padilla-Roger AM, Toma K, Herber CB, Correa SM, Duan X, Ahituv N, Tollkuhn J, Ingraham HA (2021) Oestrogen engages brain MC4R signalling to drive physical activity in female mice. Nature 599:131–135. 10.1038/s41586-021-04010-3 34646010 PMC9113400

[B16] Kurz M, Hummer M, Kemmler G, Kurzthaler I, Saria A, Fleischhacker WW (1998) Long-term pharmacokinetics of clozapine. Br J Psychiatry 173:341–344. 10.1192/bjp.173.4.341 9926040

[B17] Labuguen R, Matsumoto J, Negrete SB, Nishimaru H, Nishijo H, Takada M, Go Y, Inoue KI, Shibata T (2021) MacaquePose: a novel 'in the wild' macaque monkey pose dataset for markerless motion capture. Front Behav Neurosci 14:581154. 10.3389/fnbeh.2020.581154 33584214 PMC7874091

[B18] Lieb A, Weston M, Kullmann DM (2019) Designer receptor technology for the treatment of epilepsy. EBioMedicine 43:641–649. 10.1016/j.ebiom.2019.04.059 31078519 PMC6558262

[B19] Mimura K, Nagai Y, Inoue K, Matsumoto J, Hori Y, Sato C, Kimura K, Okauchi T, Hirabayashi T, Nishijo H, Yahata N, Takada M, Suhara T, Higuchi M, Minamimoto T (2021) Chemogenetic activation of nigrostriatal dopamine neurons in freely moving common marmosets. iScience 24:103066. 10.1016/j.isci.2021.103066 34568790 PMC8449082

[B20] Minamimoto T, La Camera G, Richmond BJ (2009) Measuring and modeling the interaction among reward size, delay to reward, and satiation level on motivation in monkeys. J Neurophysiol 101:437–447. 10.1152/jn.90959.2008 18987119 PMC2637024

[B21] Nagai Y, Kikuchi E, Lerchner W, Inoue KI, Ji B, Eldridge MA, Kaneko H, Kimura Y, Oh-Nishi A, Hori Y, Kato Y, Hirabayashi T, Fujimoto A, Kumata K, Zhang MR, Aoki I, Suhara T, Higuchi M, Takada M, Richmond BJ, et al. (2016) PET imaging-guided chemogenetic silencing reveals a critical role of primate rostromedial caudate in reward evaluation. Nat Commun 7:13605. 10.1038/ncomms13605 27922009 PMC5150653

[B22] Nagai Y, Miyakawa N, Takuwa H, Hori Y, Oyama K, Ji B, Takahashi M, Huang XP, Slocum ST, DiBerto JF, Xiong Y, Urushihata T, Hirabayashi T, Fujimoto A, Mimura K, English JG, Liu J, Inoue KI, Kumata K, Seki C, et al. (2020) Deschloroclozapine, a potent and selective chemogenetic actuator enables rapid neuronal and behavioral modulations in mice and monkeys. Nat Neurosci 23:1157–1167. 10.1038/s41593-020-0661-3 32632286

[B23] Nation HL, Nicoleau M, Kinsman BJ, Browning KN, Stocker SD (2016) DREADD-induced activation of subfornical organ neurons stimulates thirst and salt appetite. J Neurophysiol 115:3123–3129. 10.1152/jn.00149.2016 27030736 PMC4946604

[B24] Oyama K, Hori Y, Nagai Y, Miyakawa N, Mimura K, Hirabayashi T, Inoue K, Suhara T, Takada M, Higuchi M, Minamimoto T (2021) Chemogenetic dissection of the primate prefronto-subcortical pathways for working memory and decision-making. Sci Adv 7:eabg4246. 10.1126/sciadv.abg4246 34162548 PMC8221616

[B25] Paretkar T, Dimitrov E (2019) Activation of enkephalinergic (Enk) interneurons in the central amygdala (CeA) buffers the behavioral effects of persistent pain. Neurobiol Dis 124:364–372. 10.1016/j.nbd.2018.12.005 30572023 PMC6363838

[B26] Roth BL (2016) DREADDs for neuroscientists. Neuron 89:683–694. 10.1016/j.neuron.2016.01.040 26889809 PMC4759656

[B27] Soumier A, Sibille E (2014) Opposing effects of acute versus chronic blockade of frontal cortex somatostatin-positive inhibitory neurons on behavioral emotionality in mice. Neuropsychopharmacology 39:2252–2262. 10.1038/npp.2014.76 24690741 PMC4104344

[B28] Sun L, Lau CE (2000) Intravenous and oral clozapine pharmacokinetics, pharmacodynamics, and concentration-effect relations: acute tolerance. Eur J Pharmacol 398:225–238. 10.1016/s0014-2999(00)00277-6 10854834

[B29] Takano A, Varrone A, Gulyás B, Salvadori P, Gee A, Windhorst A, Vercouillie J, Bormans G, Lammertsma AA, Halldin C (2016) Guidelines to PET measurements of the target occupancy in the brain for drug development. Eur J Nucl Med Mol Imaging 43:2255–2262. 10.1007/s00259-016-3476-4 27514528 PMC5047931

[B30] Tran FH, Spears SL, Ahn KJ, Eisch AJ, Yun S (2020) Does chronic systemic injection of the DREADD agonists clozapine-N-oxide or compound 21 change behavior relevant to locomotion, exploration, anxiety, and depression in male non-DREADD-expressing mice? Neurosci Lett 739:135432. 10.1016/j.neulet.2020.135432 33080350

[B31] Upright NA, Baxter MG (2020) Effect of chemogenetic actuator drugs on prefrontal cortex-dependent working memory in nonhuman primates. Neuropsychopharmacology 45:1793–1798. 10.1038/s41386-020-0660-9 32193513 PMC7608232

[B32] Upright NA, Brookshire SW, Schnebelen W, Damatac CG, Hof PR, Browning PG, Croxson PL, Rudebeck PH, Baxter MG (2018) Behavioral effect of chemogenetic inhibition is directly related to receptor transduction levels in Rhesus monkeys. J Neurosci 38:7969–7975. 10.1523/JNEUROSCI.1422-18.2018 30082415 PMC6136156

[B33] Urban DJ, Roth BL (2015) DREADDs (designer receptors exclusively activated by designer drugs): chemogenetic tools with therapeutic utility. Annu Rev Pharmacol Toxicol 55:399–417. 10.1146/annurev-pharmtox-010814-124803 25292433

[B34] Wakaizumi K, Kondo T, Hamada Y, Narita M, Kawabe R, Narita H, Watanabe M, Kato S, Senba E, Kobayashi K, Kuzumaki N, Yamanaka A, Morisaki H, Narita M (2016) Involvement of mesolimbic dopaminergic network in neuropathic pain relief by treadmill exercise: a study for specific neural control with Gi-DREADD in mice. Mol Pain 12:1744806916681567. 10.1177/1744806916681567 27909152 PMC5140073

[B35] Weston M, Kaserer T, Wu A, Mouravlev A, Carpenter JC, Snowball A, Knauss S, von Schimmelmann M, During MJ, Lignani G, Schorge S, Young D, Kullmann DM, Lieb A (2019) Olanzapine: a potent agonist at the hM4D(Gi) DREADD amenable to clinical translation of chemogenetics. Sci Adv 5:eaaw1567. 10.1126/sciadv.aaw1567 31001591 PMC6469940

[B36] Yan X, Telu S, Dick RM, Liow JS, Zanotti-Fregonara P, Morse CL, Manly LS, Gladding RL, Shrestha S, Lerchner W, Nagai Y, Minamimoto T, Zoghbi SS, Innis RB, Pike VW, Richmond BJ, Eldridge MA (2021) [11C]Deschloroclozapine is an improved PET radioligand for quantifying a human muscarinic DREADD expressed in monkey brain. J Cereb Blood Flow Metab 41:2571–2582. 10.1177/0271678X211007949 33853405 PMC8504956

[B37] Yu S, Münzberg H (2018) Testing effects of chronic chemogenetic neuronal stimulation on energy balance by indirect calorimetry. Bio Protoc 8:e2811. 10.21769/BioProtoc.2465 PMC6018017 29951570

[B38] Zhou XT, Bao WD, Liu D, Zhu LQ (2020) Targeting the neuronal activity of prefrontal cortex: new directions for the therapy of depression. Curr Neuropharmacol 18:332–346. 10.2174/1570159X17666191101124017 31686631 PMC7327942

[B39] Zou D, Chen L, Deng D, Jiang D, Dong F, McSweeney C, Zhou Y, Liu L, Chen G, Wu Y, Mao Y (2016) DREADD in parvalbumin interneurons of the dentate gyrus modulates anxiety, social interaction and memory extinction. Curr Mol Med 16:91–102. 10.2174/1566524016666151222150024 26733123 PMC4997952

